# Supplementary material for: Cortical markers of PAS-induced long-term potentiation and depression in the motor system: A TMS-EEG Registered Report
Source: Imaging Neurosci (Camb). 2026 Jan 9;4:IMAG.a.1086. doi: 10.1162/IMAG.a.1086 (PMC12794311; doi:10.1162/IMAG.a.1086)
Supplement: Supplementary Material [file IMAG.a.1086_supp.pdf]

Cortical markers of PAS-induced long-term potentiation and depression in the motor system: A TMS-EEG Registered Report

Eleonora Arrigoni, Nadia Bolognini, Alberto Pisoni & Giacomo Guidali

– SUPPLEMENTARY MATERIALS –

Protocol	rMT	110% condition and PAS	90% condition	sensory threshold (electrical stimulation)	PAS electrical stimulation intensity
**PAS$_{LTP}$**	43.9 ± 8.5%	48.4 ± 9.3%	39.1 ± 7.6%	3.16 ± 0.83 mA	9.49 ± 2.37 mA
**PAS$_{LTD}$**	43.3 ± 8.6%	47.6 ± 9.5%	38.3 ± 7.5%	3.14 ± 0.66 mA	9.09 ± 2.26 mA

Supplemental Table S1. TMS and electrical stimulator's mean ± standard deviation – SD of parameters used in the experimental sessions.

		**PAS$_{LTP}$**					**PAS$_{LTD}$**				
		**110%**			**90%**		**110%**			**90%**	
		**T0**	**T1**	**T2**	**T0**	**T1**	**T0**	**T1**	**T2**	**T0**	**T1**
**ICA components removed**	***Mean***	2,07	2,10	2,17	2,20	2,03	2,07	2,23	2,07	2,13	2,20
	SD	1,08	0,76	0,75	1,13	0,67	1,05	0,94	0,98	0,82	0,81
**SSP-SIR components removed**	***Mean***	2,90	2,79	2,86	1,97	1,90	2,83	2,79	2,72	2,17	2,24
	SD	0,88	0,76	0,86	1,02	1,03	0,76	0,76	0,64	1,00	1,11

Supplemental Table S2. Mean ± SD number of ICA and SSP-SIR components removed during EEG preprocessing in the different experimental sessions.

Variable	Protocol	Comparison	Statistics
**(log-transformed) MEP**	PAS$_{LTP}$	T2 *vs.* T0	$t_{29} = 2.68$, $p_{tukey} = .11$
		T2 *vs.* T1	$t_{29} = -2.24$, $p_{tukey} = .25$
	PAS$_{LTD}$	T2 *vs.* T0	$t_{29} = -2.25$, $p_{tukey} = .245$
		T2 *vs.* T1	$t_{29} = 0.3$, $p_{tukey} = .99$
**(log-transformed) P30**	PAS$_{LTP}$	T2 *vs.* T0	$t_{29} = 2.12$, $p_{tukey} = .304$
		T2 *vs.* T1	$t_{29} = -2.14$, $p_{tukey} = .297$

	PAS_{LTD}	T2 *vs.* T0	t_{29} = -1.47, p_{tukey} = .687
		T2 *vs.* T1	t_{29} = -0.67, p_{tukey} = .984

Supplemental Table S3. T2 *vs.* T0 and T1 post-hoc comparisons for statistically significant 'PAS protocol' X 'Time' interactions found for **H3** analyses.

Variable	Factor	Statistics
**(log-transformed) P30**	PAS protocol	$F_{1,19}$ = 0.03, p = .867, η_p^2 < .01
	Time	$F_{1,19}$ = 0.5, p = .489, η_p^2 = .03
	PAS protocol X Time	$F_{1,19}$ = 0.07, p = .799, η_p^2 < .01
**(log-transformed) P60**	PAS protocol	$F_{1,19}$ = 0.75, p = .396, η_p^2 = .04
	Time	$F_{1,19}$ = 0.03, p = .864, η_p^2 < .01
	PAS protocol X Time	$F_{1,19}$ < 0.01, p = .99, η_p^2 < .01
**(log-transformed) N100**	PAS protocol	$F_{1,19}$ = 0.18, p = .674, η_p^2 = .01
	Time	$F_{1,19}$ = 0.22, p = .647, η_p^2 = .01
	PAS protocol X Time	$F_{1,19}$ = 1.5, p = .235, η_p^2 = .07

Supplemental Table S4. Results from the rmANOVAs conducted on M1-TEP peaks extracted in the 90% rMT conditions.

Dependent variable	Predictor	Generalized linear regression model fit
**MEP *PAS_{LTP} effect***	rMT	R^2 = 0.01, χ^2_1 = 0.21, p = .628
	(log-transformed) MEP	R^2 < 0.01, χ^2_1 = 0.16, p = .675
	(log-transformed) P30	R^2 < 0.01, χ^2_1 = 0.1, p = .74
	P60	R^2 = 0.01, χ^2_1 = 0.21, p = .629
	N100	R^2 = 0.14, χ^2_1 = 3.6, p = .031
**MEP *PAS_{LTD} effect***	rMT	R^2 = 0.01, χ^2_1 = 0.04, p = .666
	**(log-transformed) MEP**	**R^2 = 0.17, χ^2_1 = 1.05, p = .015**
	(log-transformed) P30	R^2 = 0.02, χ^2_1 = 0.11, p = .463
	P60	R^2 = 0.03, χ^2_1 = 0.19, p = .339
	N100	R^2 < 0.01, χ^2_1 < 0.01, p = .955
**P30 *PAS_{LTP} effect***	rMT	R^2 < 0.01, χ^2_1 = 0.02, p = .864
	(log-transformed) MEP	R^2 = 0.01, χ^2_1 = 0.22, p = .568
	(log-transformed) P30	R^2 = 0.11, χ^2_1 = 2.02, p = .068
	**P60**	**R^2 = 0.32, χ^2_1 = 12.99, p < .001**

	N100	$R^2 = 0.04$, $\chi^2_1 = 0.69$, $p = .306$
**N100 *PAS$_{LTD}$ effect***	rMT	$R^2 = 0.01$, $\chi^2_1 = 0.01$, $p = .623$
	(log-transformed) MEP	$R^2 < 0.01$, $\chi^2_1 < 0.01$, $p = .956$
	(log-transformed) P30	$R^2 = 0.11$, $\chi^2_1 = 0.11$, $p = .069$
	P60	$R^2 < 0.01$, $\chi^2_1 < 0.01$, $p = .813$
	N100	$R^2 = 0.01$, $\chi^2_1 = 0.01$, $p = .578$

Supplemental Table S5. Results from the generalized linear regression models (function: Gaussian, link: Identity) ran to explore the predictability of T0 variables for PAS aftereffects (i.e., ratio of T1 values over T0). Significant ($p < .02$) effects are reported in bold.

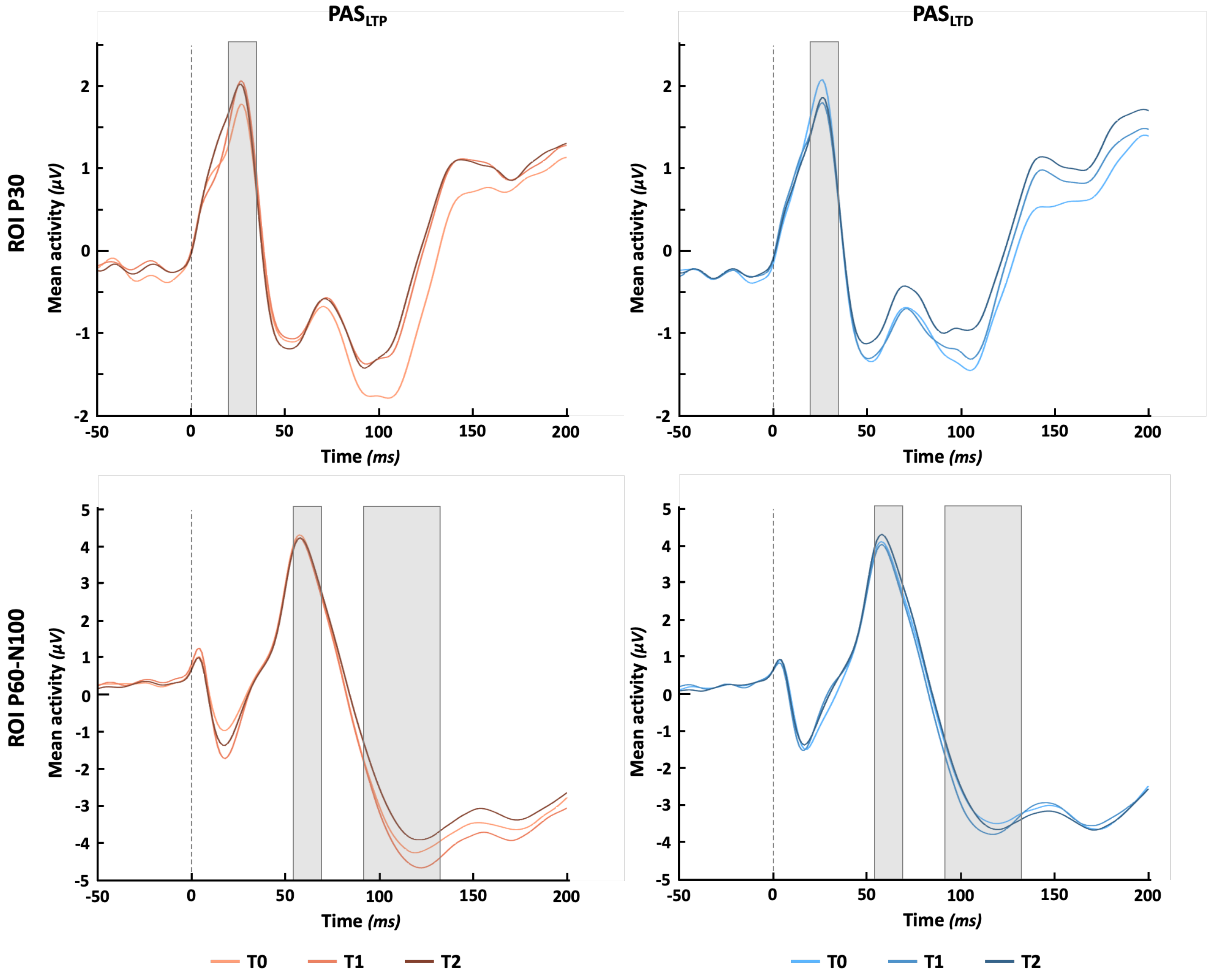

Supplemental Figure S1. Mean activity in the two ROIs selected for peak extraction (upper panels: P30 ROI: Cz, C2, CP2, CP4 electrodes, lower panels: P60 and N100 ROI: CP1, CP3, CP5, P3 electrodes) in 110% rMT conditions. Grey-shaded areas over the plots show time windows of P30, P60, and N100 components extraction.

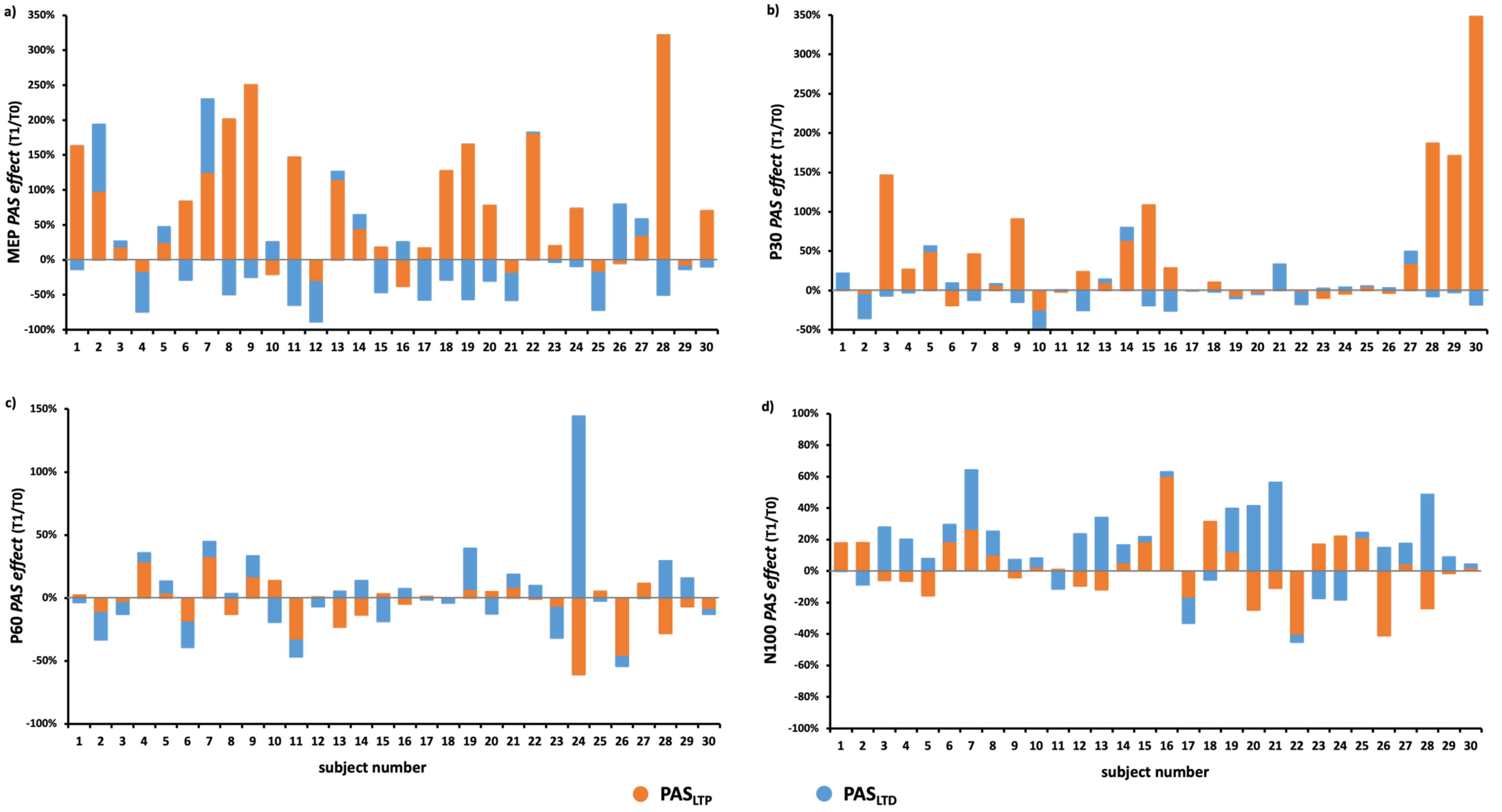

Supplemental Figure S2. *PAS effect* (i.e., the ratio between T1 and T0 amplitude) for MEPs (**a**), P30 (**b**), P60 (**c**), and N100 (**d**) components at the single-subject level according to the two protocols (orange bars: PAS_{LTP}, blue bars: PAS_{LTD}).
